# Cardiac Resynchronization Therapy in Pediatrics

**DOI:** 10.19102/icrm.2018.090804

**Published:** 2018-08-15

**Authors:** Allison C. Hill, Michael J. Silka, Yaniv Bar-Cohen

**Affiliations:** ^1^Division of Cardiology, Children’s Hospital Los Angeles, Los Angeles, CA, USA; ^2^Keck School of Medicine, University of Southern California, Los Angeles, CA, USA

**Keywords:** Cardiac resynchronization therapy, heart failure, pediatrics

## Abstract

Cardiac resynchronization therapy (CRT) has proven to be a powerful and effective tool in the treatment of adults with severe dilated or ischemic cardiomyopathy. A substantial portion of the adult heart failure population has severely depressed systolic function, heart failure symptoms, QRS prolongation, and left bundle branch block. Indications for CRT in adults are commonly focused on these characteristics. However, pediatric patients represent a heterogeneous group with many etiologies of heart failure and anatomic variants, with most of them not fitting the typical adult CRT criteria. The heterogeneity of the pediatric population has hindered the identification of ideal candidates for CRT, but initial experience with CRT in various groups of pediatric patients has been encouraging. This article reviews indications for and outcomes of CRT in pediatric and congenital heart disease patients.

## Introduction

Heart failure is a common medical problem that affects 2% to 3% of the general adult population and an estimated 12,000 to 35,000 children per year.^1,2^ While a number of different etiologies can be responsible for heart failure, a subset of heart failure patients have cardiac dyssynchrony, defined as ventricular contraction that does not occur in the usual organized sequence. This dyssynchronous ventricular contraction can be due to the electrical activation of the ventricle not propagating through the insulated His-Purkinje system.^3^ The results of electrical dyssynchrony can include decreased efficiency of ventricular contraction, increased atrioventricular valvar regurgitation, and abnormal ventricular remodeling.

When dyssynchrony is suspected in heart failure patients, one approach to heart failure therapy is restoring synchrony via cardiac resynchronization therapy (CRT). This can be accomplished using a variety of pacing maneuvers, with the most frequent one used being biventricular pacing. By reestablishing cardiac synchrony, hemodynamics may be improved due to the optimization of the interventricular and intraventricular contraction sequences, thereby potentially decreasing mitral regurgitation, reducing pulmonary venous pressure, increasing myocardial preload, and improving cardiac output.^4,5^ However, while a large body of clinical experience and literature has accumulated regarding CRT in adult patients, there is significantly less known about CRT in pediatric populations.

## Cardiac resynchronization therapy in adult populations

CRT has been used most frequently in adult dilated cardiomyopathy (DCM) patients with severe systolic dysfunction [left ventricular ejection fraction (LVEF) ≤ 35%] and left bundle branch block (LBBB), with its use benefiting a substantial number of these individuals.^2^ Multiple large clinical trials have shown significant improvements in LVEF within months of CRT initiation: the Cardiac Resynchronization—Heart Failure (CARE-HF) study, for example, showed an increase in LVEF by 4% within three months, while the Multicenter InSync Randomized Clinical Evaluation (MIRACLE) trial demonstrated a 5% improvement within six months following CRT initiation.^6,7^ These improvements were accompanied by enhancements in long-term outcomes such as chronic exercise tolerance and heart failure symptoms and a significant reduction in mortality.^8–12^

Because potential benefits for individual patients are clear, significant efforts have been made to identify the ideal candidates for CRT, especially since approximately 30% of adult patients are nonresponders to CRT. One determinant of optimal response to CRT is QRS duration: CRT is associated with a significant benefit in adults with a QRS duration ≥ 150 ms, but not in those with a QRS duration < 150 ms.^13,14^ A second determinant of response to CRT is QRS morphology: patients with a LBBB morphology are more likely to derive benefit from CRT than those with a non-LBBB morphology.^15,16^ Third, the benefit of CRT may not be as dependent on the severity of symptoms in comparison with other factors, since both patients with advanced heart failure and patients with mild heart failure symptoms have been shown to derive benefit from CRT.^3,12–14^ Thus, guidelines for CRT in adults focus on LVEF ≤ 35%, the presence of a LBBB pattern, a significantly prolonged QRS duration (QRS duration ≥ 150 ms), and the onset of at least mild symptomatic heart failure [New York Heart Association (NYHA) functional classes II, III, or IV], as shown in **Table 1**.^17,18^ However, in the setting of severe heart failure and heart block with an anticipated need for frequent ventricular pacing (> 40% of the time), CRT has been deemed appropriate regardless of symptoms or intrinsic QRS duration.^19^

## Cardiac resynchronization therapy in pediatric and congenital heart disease populations

The efficacy of CRT in the adult population has been encouraging and has led to efforts to identify potential applications in pediatric patients. However, the pediatric heart failure population is heterogeneous in both anatomy and etiology of heart failure; thus, the adult experience cannot easily be applied in pediatrics. To date, a small number of studies have examined the potential benefits and outcomes of CRT in a variety of pediatric patient groups, including those with normal anatomy and those with congenital heart disease (CHD).

Dubin et al. described a large cohort of pediatric and adult CHD patients in which 103 had CRT devices implanted (median age: 12.8 years).^20^ This cohort included 73 patients (71%) with CHD, 16 (15.5%) with cardiomyopathy, and 14 (13.5%) with congenital complete heart block. Almost half (45%) of these individuals had pacemakers prior to the CRT devices. Over the follow-up period (mean: 4.5 months), the QRS duration improved by 38 ms ± 31 ms (from 166 ms ± 33 ms to 126 ms ± 24 ms; p < 0.01) and the EF improved by 14% ± 13% (from 26% ± 12% to 40% ± 15%; p < 0.05). Improvements in QRS duration and EF were seen in all three groups, with no significant differences between the outcomes between them. Eleven percent of the cohort were nonresponders, defined as those with either worsening or no change in EF following CRT placement. Nonresponders had a higher baseline EF (32% ± 14.2% versus 24.3% ± 11%; p = 0.04) but no other significant differences between them and those who responded to CRT.

Janoušek et al.^21^ described a multicenter cohort of 109 CRT patients with a greater proportion of CHD patients (80%) as compared with those in the study by Dubin et al.^20^ Most of the patients in this cohort (77%) had dyssynchrony associated with single-site pacing, although 23% had electrical dyssynchrony with intrinsic atrioventricular nodal conduction. Of these, 9% had LBBB with a systemic LV, 5% had right bundle branch block (RBBB) with a systemic right ventricle (RV) or single ventricle, and 9% had nonspecific QRS prolongation. During follow-up (median: 7.5 months), similar improvements in QRS duration (median: 40 ms improvement from a starting median QRS duration of 160 ms) and EF (median: 12% improvement from a median starting EF of 27%) were seen. Janoušek et al.^21^ also found a larger portion of the population (19%) were nonresponders versus those identified by Dubin et al.^20^ and ascertained DCM and poor baseline NYHA functional class as the best predictors of nonresponse. Furthermore, in their study, the best predictor of improvement in cardiac function following CRT was the presence of a systemic LV.

These two CRT studies demonstrate the heterogeneity of the pediatric heart failure population, both in terms of anatomy and etiology of heart failure, and the limited value of extrapolating adult CRT data to pediatric patients. Based on the heterogeneity of the pediatric heart failure population, there is no current consensus on timing or patient selection guidelines for CRT in pediatrics at this time. However, some patterns have been identified based on the studies available.

### Cardiac resynchronization therapy for dilated cardiomyopathy in pediatrics

Pediatric patients with LV failure often have a primary cardiomyopathy, while a dyssynchronous ventricular contraction can at times be visualized on echocardiography. This mechanical dyssynchrony is not always associated with electrical dyssynchrony (sometimes defined by QRS duration z-score > 2).^22,23^ In fact, while 65% of pediatric patients with idiopathic DCM exhibit mechanical dyssynchrony, QRS duration is generally normal in this group and similar to in those without mechanical dyssynchrony (mean QRS duration of 87 ms and 85 ms, respectively, for pediatric patients with and without mechanical dyssynchrony).^22^ The lack of correlation between mechanical and electrical dyssynchrony in pediatric patients makes patient selection for CRT difficult, even in the pediatric population of DCM patients who would otherwise seem to be most similar to classic adult CRT responders. In addition, a LBBB morphology is only present in 0% to 9% of pediatric heart failure patients.^22–24^ This is substantially less than the 25% of adults with heart failure who have a LBBB.^25^ The infrequency with which pediatric patients with LV failure have a prolonged QRS or a LBBB means that the large majority of pediatric patients with LV failure do not meet standard adult criteria for CRT. In fact, when Schiller et al. reviewed all DCM patients at a large pediatric center, none of the patients met adult class I criteria for CRT.^23^ Therefore, the group of pediatric patients who have met standard adult criteria is small and only represents a small portion of pediatric CRT patients (2%–9%).^24^

When CRT has been utilized in the pediatric DCM population, improvements in QRS duration and EF have been observed. In 10 patients with DCM studied by Janoušek et al., the median QRS duration decreased by 14 ms (from a median starting QRS duration of 144 ms), while an 11% increase in the EF was demonstrated.^21^ Despite these changes in QRS duration and EF, however, the patients in this study did not show any improvement in their NYHA classification from pre-CRT (at which time, the median NYHA functional class was IV) to post-CRT, and six of nine patients (one patient did not have follow-up data) were deemed nonresponders despite the overall improvements in QRS duration and EF. The authors concluded that one of the only independent multivariable predictors for nonresponse to CRT was the presence of primary DCM. In comparison, in the study by Dubin et al.,^20^ 16 primary cardiomyopathy patients (mean age: 15.8 years) also demonstrated a marked narrowing of the QRS complex (mean reduction: 32 ms) and an improvement in EF (mean: 12.3%).

### Cardiac resynchronization therapy for pacing-induced cardiomyopathy

Another important group of pediatric patients with failing LVs is comprised of those with heart block who develop pacemaker-induced cardiomyopathy. In chronically-paced pediatric patients, the dyssynchrony caused by pacing from a single site (especially when from the RV) results in a subset (13%) of patients later manifesting a cardiomyopathy.^26^ Specifically, pacing the RV outflow tract or lateral RV has predicted a depressed LV function (EF < 45%) with an odds ratio of 10.7, whereas pacing at the LV apex or midlateral LV wall predicts a preserved LV function (EF ≥ 55%) with an odds ratio of 8.3.^27^ Thus, patients who have permanent pacemaker leads on the RV outflow tract or lateral RV may develop heart failure that could theoretically be reversed by CRT. In fact, chronically paced heart block patients represent a large proportion (45%–78%) of pediatric patients who have undergone CRT.^21,28^

**Figure 1:**
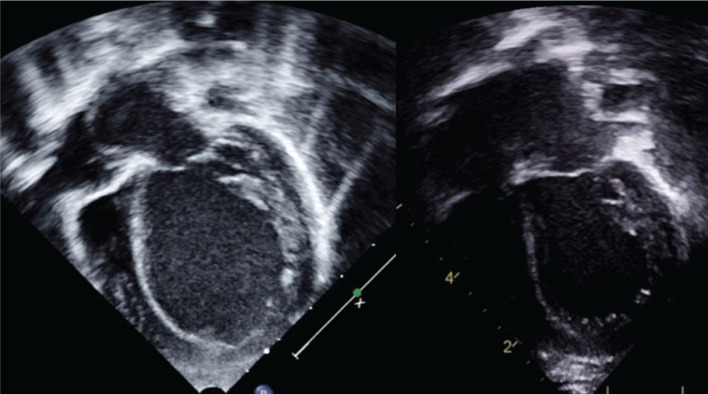
Pacemaker-induced cardiomyopathy in a four-month-old with congenital atrioventricular block before and at two weeks after a left ventricular lead was added.

Significant improvements in QRS duration (by 37–40 ms) with CRT have been reported in pacemaker-induced cardiomyopathy patients, potentially because their initial paced QRS durations tend to be quite prolonged (median: 155 ms).^20,21^ These patients also demonstrate a 16% to 23% improvement in EF.^20,21^ In contrast with DCM patients, pacemaker-induced cardiomyopathy patients have shown a consistent improvement in clinical status with CRT, with Janoušek et al. reporting an improvement in median NYHA functional class by one level (from a baseline of class II) and noting that a relatively small proportion of this group are nonresponders (16%).^21^ The apparent superiority of CRT in pacemaker-mediated cardiomyopathy over other pediatric cardiomyopathies may be related to the relatively normal individual myocyte function in already-paced patients with dyssynchrony, whereas intrinsic myocyte dysfunction is less likely to be reversible in the setting of other advanced cardiomyopathies. **Figure 1** demonstrates the dramatic improvement in LV size within two weeks of CRT implantation in a four-month-old patient with pacemaker-induced cardiomyopathy.

Tetralogy of Fallot (TOF) patients can also develop LV failure (at rates reported to be up to 5%–10%).^29,30^ In a small series of 10 adults with TOF, LV dysfunction and electrical dyssynchrony (QRS duration: 182 ms ± 35 ms; four had atrioventricular nodal conduction and RBBB, and six had heart block and were paced from the RV apex), CRT was reported to improve LVEF from 24% ± 11% to 37% ± 13% over a period of nine months.^31^ Case reports of pediatric TOF patients with LV failure have also described improvement in hemodynamics both acutely and in the longer-term. Biventricular stimulation led to improved LV function and successful weaning off of extracorporeal circulation in a six-month-old infant with TOF.^32^ In the case of a five-year-old chronically RV-paced TOF patient with progressive LV dysfunction, biventricular pacing resulted in an improvement in symptoms by four months post-CRT implantation, with marked improvement in LV systolic function.^33^

### Cardiac resynchronization therapy for subpulmonary right ventricular failure

Since CRT has been shown to improve LV dysfunction in the setting of LBBB in adults, it has been proposed that CRT could potentially be used in patients with subpulmonary RV dysfunction in the setting of RBBB. The majority of this population is made up of patients with TOF in whom electrical dyssynchrony and a long QRS (with RBBB) has developed either due to scarring on the RV from surgical repairs or from chronic pressure or volume overload related with pulmonary valve dysfunction. In addition, patients with aortic stenosis status following a Ross procedure or those with pulmonary hypertension can also develop RV failure with RBBB.

The efficacy of CRT has been studied in both acute and longer-term settings, although the patient numbers considered have been small. Crucially, pacing patients with RBBB at different RV sites or in conjunction with LV pacing (to achieve biventricular pacing) has been found to acutely increase the systemic blood pressure and decrease the QRS duration.^34^ In addition, acutely pacing patients with RBBB at different sites (RV inflow, RV apex, and RVOT) demonstrated that the pacing site with the narrowest QRS was associated with the greatest improvement in cardiac index.^35^

Longer-term studies on CRT in patients with RBBB are even rarer. Biventricular pacing for CRT in nine adult TOF patients with RV failure was reported to improve global activation time and dyssynchrony index. In addition, biventricular pacing resulted in improved NYHA classification, exercise tolerance, and LVEF (from 50% to 56%; p = 0.02).^36,37^ The larger pediatric CRT series (Janoušek et al.^21^ and Dubin et al.^20^) included 11 and six patients with TOF, respectively. Although the results were generally favorable for all CRT patients in those studies, outcomes were not specifically reported for patients with RBBB or TOF.^20,21,24^ Thus, while small studies have shown some early promising results in the acute and short-term follow-up period for patients with TOF and RBBB, larger and longer-term studies have not been reported and predictors of optimal response, best pacing location, and ideal pacing timing are not clear.

### Cardiac resynchronization therapy for systemic right ventricular failure

Patients with congenitally-corrected transposition of the great arteries (CCTGA) and those with TGA status following atrial switch frequently develop systemic RV failure, especially after entering adulthood.^38,39^ These patients can develop RV failure and dyssynchrony due to myocardial scarring and/or chronic pressure overload and have been considered for CRT. In addition, patients with CCTGA can develop atrioventricular block and ventricular dysfunction and may be particularly interesting as candidates for CRT.

Although limited, some data exist for patients with systemic RV failure, such as those with CCTGA or TGA status following atrial switch, who have undergone CRT. CRT in this population has been shown to decrease QRS duration and increase the EF of the systemic RV.^20,21,40^ However, the change in clinical status has not been as clear: clinical improvement has been seen among a broad range of patients (in as few as 25% and as many as 100%).^20,21,24^ The mixed results may be related to the older age at which patients in this population have tended to undergo CRT, which could affect the reversibility of damage to the myocardium.^24^

It is also important to note that, while patients with systemic RVs can develop significant systemic atrioventricular valve regurgitation, CRT has not been shown to reduce the degree of atrioventricular valve regurgitation in this population. Overall, results of CRT in patients with systemic RV failure are mixed and determinants of optimal responders in this special population have not been clearly identified.

### Cardiac resynchronization therapy for single-ventricle failure

Single-ventricle failure is likely to occur due to myocardial fibrosis from cardiac surgeries, chronic volume and/or pressure overload, or abnormal ventricular morphology. Intraventricular resynchronization by multisite pacing has been used in an attempt to improve ventricular synchrony and function in these individuals. CRT in this setting has decreased QRS duration, but clinical improvements have been variable. Some small studies have reported as many as 91% of single-ventricle patients deriving clinical improvement from CRT, while others describe only 26% having clinical benefit.^20,21,24^

Thus, like in several other pediatric CRT groups, a decrease in QRS duration is not necessarily correlated with a clinical improvement in this population. One potential reason for this is the lack of correlation between electrical and mechanical dyssynchrony in single-ventricle physiology. Motonaga et al. described 11 patients with hypoplastic left heart syndrome as having marked mechanical dyssynchrony as measured by echocardiographic tissue Doppler imaging and vector velocity imaging.^41^ Indices of mechanical dyssynchrony were then compared with an electrical dyssynchrony index created with three-dimensional electroanatomic mapping. Despite the presence of markedly abnormal mechanical dyssynchrony indices, electrical dyssynchrony indices were not different from normal controls and the mechanical dyssynchrony index did not correlate with the electrical dyssynchrony index in this patient cohort. While there appear to be some single-ventricle patients who derive clinical improvement from CRT, criteria for identifying responders remain elusive.

## Technical considerations

### Lead placement

**Figure 2:**
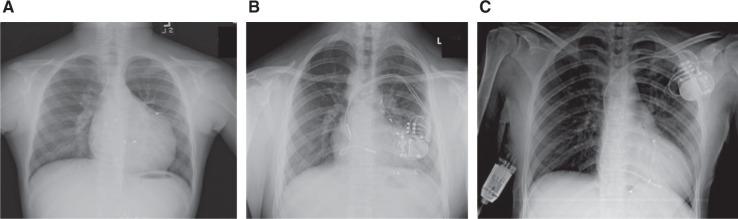
Chest X-rays showing variations of configurations for CRT in pediatric patients. **A:** Epicardial configuration. **B:** Transvenous configuration. **C:** Hybrid approach with LV lead tunneled from the epicardium.

Approximately half of CRT devices in pediatric patients have been implanted via a transvenous approach.^20^ The other half necessitate epicardial placement either due to (1) the presence of CHD with intracardiac shunting, venous anomalies, tricuspid valve abnormalities, or other anatomic abnormalities incompatible with a transvenous system; or (2) a small size such that a transvenous system is not advised.^20^ A hybrid approach may also be employed to upgrade a current transvenous system with an epicardial LV lead for CRT **(Figure 2)**. CRT lead placement depends on the physiologic and anatomic substrate: while most CRT involves biventricular pacing, single-site pacing may suffice for CRT in some pediatric patients, including those with an RBBB morphology. The optimization of RV electrical activation can theoretically be achieved by pacing the RV with an optimal atrioventricular interval such that the paced wavefront merges with the intrinsic wavefront from the left bundle or left heart. This approach aims to shorten the QRS duration and to achieve a more synchronous electrical activation. While some data support this,^35^ other small studies have found that the optimal RV pacing site varied between patients and that the site that resulted in the narrowest QRS complex was not necessarily the site that correlated with the best RV performance.^34,35,42^ Thus, the optimal site of RV lead placement (as well as physiological benefit) for single-site pacing in the setting of RBBB is not entirely clear.

### Cardiac resynchronization therapy implantation in single-ventricle patients

Lead placement in patients with single-ventricle anatomy aims to achieve intraventricular synchrony as opposed to interventricular synchrony as is the case in biventricular anatomy. With intraventricular synchrony as a goal, identifying the optimal location for lead placement can be limited by the lack of techniques for evaluating synchrony (especially intraoperatively at the time of lead placement). In addition, prior surgical scars or other causes of ventricular scarring may impact the capture thresholds for potential lead locations, resulting in limited potential pacing sites. Lastly, anatomic variations and a need to avoid coronary arteries limit optimal lead placement in the single-ventricle population.

### Evaluation of dyssynchrony

One of the primary challenges in identifying candidates for pediatric CRT is the technical challenge of evaluating dyssynchrony. This is especially difficult when considering the lack of a direct correlation between electrical and mechanical dyssynchrony that is often seen in pediatrics. In a study of pediatric patients with DCM, those with mechanical dyssynchrony had similar QRS durations as compared with those considered to have a mechanically synchronous contraction.^22^ However, when both mechanical and electrical dyssynchrony are suspected, recent evidence in CHD patients suggests that an increase in dP/dt max of at least 15% due to CRT use correlates with an improvement in NYHA classification.^43^ As techniques to detect and quantify mechanical and electrical dyssynchrony improve, other acute surrogates of contractility response, in addition to dP/dt max testing, may play a role in identifying optimal candidates for CRT.

## Safety

The overall adverse event rate in pediatric CRT studies has been 10% to 29%.^20,21,24^ The most common adverse event is coronary sinus lead issues (18% of all patients with transvenous CRT devices), such as dislodgement, difficulty placing the lead, and phrenic stimulation, with generally minimal sequelae. However, more severe complications, including death (5%–8%) and malignant ventricular arrhythmias, have been reported.^44^ While some pediatric patients who undergo CRT have complex CHD, the mortality rate does not appear to be related to procedural difficulty due to patient anatomy but rather to the progression of their intrinsic cardiovascular disease or to the presence of malignant ventricular arrhythmias following implantation.^20,21,24^ The proarrhythmia effect of CRT in pediatrics has not been thoroughly described, but two of the 103 patients in the study of Dubin et al. and two of the 109 patients in the study of Janoušek et al. were reported to have ventricular tachycardia or fibrillation.^20,21^ These complication rates are comparable to those in adult CRT studies.^6,12^

## Defining indications for pediatric cardiac resynchronization therapy

In contrast with adults, where those with idiopathic or ischemic cardiomyopathy with secondary dyssynchrony are the individuals most frequently targeted for CRT, the clearest indication for CRT in pediatric patients appears to be in the setting of pacemaker-induced cardiomyopathy. This population has been shown to have reverse remodeling with improvements in EF and clinical symptoms. Pediatric patients with primary DCM do not demonstrate as clear of an indication for CRT, likely secondary to the lack of correlation between mechanical and electrical dyssynchrony and the relative rarity of defined electrical dyssynchrony in this population. There are insufficient long-term data to predict which patients with CHD—including those with TOF, single ventricles, or systemic right ventricles—would benefit the most from CRT. **Table 2** summarizes the outcomes of CRT by disease process.

## Future considerations

Recently, there has been renewed interest in the use of direct stimulation of the His bundle as an attempt to achieve the most physiologic synchronous ventricular stimulation. Although this method was initially proposed in 1967,^45^ reports of clinical attempts to achieve synchronous ventricular activation via His-bundle pacing have only actively been pursued in the current decade.^46–48^ In part, this is based on an improved understanding of the proximal conduction system and its relationship with the atrioventricular septum.^49^ Optimizing methods to avoid direct myocardial stimulation and instead selectively pace the His bundle, which is surrounded by fibrous tissue as it traverses the membranous ventricular septum, is an area of current active investigation. While early results have been encouraging, reported data have been confined to adult patients with structurally normal hearts. Though selective His-bundle pacing may ultimately require a novel design of the pacing electrode and delivery system, direct stimulation of the conduction system may provide a major opportunity to improve the outcome of young patients, particularly those with CHD who require lifelong cardiac pacing.

Multisite pacing is also a more recent area of investigation in adult CRT patients. Multisite pacing focuses on the delivery of cardiac stimulation from a number of sites in the LV and RV with the goal of achieving a more synchronous overall cardiac activation. Although studies of adult patients have suggested the potential for this modality,^50^ investigations in pediatric and CHD patients remain rudimentary.^51^

## Conclusions

CRT has been demonstrated to be an effective treatment option in adult heart failure patients, but very few pediatric patients meet the traditional adult criteria for CRT of a low EF and a significantly prolonged QRS duration from a LBBB. While there appears to be a clear role for CRT in pacemaker-induced cardiomyopathy, there is no consensus on indications for CRT in other pediatric populations. The role of CRT in CHD patients with RV failure and a RBBB, those with a systemic RV, and those who are single-ventricle patients, is yet to be defined and is challenged by the paucity of available data.

## Figures and Tables

**Table 1:** Indications for CRT in Adults^14^

Recommendation	Recommendation Classification	Level of Evidence
Indicated if patient demonstrates LVEF ≤ 35%; sinus rhythm; LBBB with QRSd ≥ 150 ms; and NYHA class II, III, or ambulatory IV symptoms on guidelines-directed medical therapy	I	A for NYHA class III/VI; B for NYHA class II
Can be useful in patients who have an LVEF ≤ 35%; sinus rhythm; non-LBBB with a QRSd ≥ 150 ms; and NYHA functional class III or ambulatory IV symptoms on guidelines-directed medical therapy	IIa	A
Can be useful in patients who have an LVEF ≤ 35%; sinus rhythm; non-LBBB with a QRSd of 120 ms–149 ms; and NYHA class II, III, or ambulatory IV symptoms on guidelines-directed medical therapy	IIa	B
Can be useful in patients who have AF and an LVEF ≤ 35% on guidelines-directed medical therapy if they require ventricular pacing or otherwise meet CRT criteria and when atrioventricular nodal ablation or pharmacologic rate control will allow for near-100% ventricular pacing with CRT	IIa	B
Can be useful if the patient demonstrates LVEF ≤ 35% on guidelines-directed medical therapy and if they are undergoing new or replacement device implantation with an anticipated significant (> 40%) requirement of ventricular pacing	IIa	C
May be considered if the patient demonstrates LVEF ≤ 35%; sinus rhythm; a non-LBBB pattern with a QRSd 120 ms to 149 ms; and NYHA class III/ambulatory IV on guidelines-directed medical therapy	IIb	B
May be considered if the patient demonstrates LVEF ≤ 35%; sinus rhythm; a non-LBBB pattern with a QRSd ≥ 150 ms; and NYHA functional class II on guidelines-directed medical therapy	IIb	B
May be considered if the patient demonstrates LVEF ≤ 30%; ischemic heart failure; sinus rhythm; LBBB pattern with a QRSd ≥ 150 ms; and NYHA functional class I on guidelines-directed medical therapy	IIb	C
Not recommended for patients with NYHA functional class I or II and/or those with a non-LBBB pattern with a QRSd < 150 ms	III	B
Not indicated in patients whose comorbidities and/or frailty limit their chances of survival with good functional capacity to a period of less than one year	III	C

LVEF: left ventricular ejection fraction; LBBB: left bundle branch block; QRSd: QRS duration; NYHA: New York Heart Association; AF: atrial fibrillation; CRT: cardiac resynchronization therapy.

**Table 2:** Outcomes of CRT in Pediatrics by Underlying Disease^17,18,21,33,34,37,42^

	Improvement in QRS Duration	Improvement in EF	Clinical Improvement	Nonresponders
DCM with LBBB	14–32 ms	11%–12%	No change according to limited data	67%
Pacemaker-induced cardiomyopathy	37–40 ms	16%–23%	NYHA 2.5 to 1.5	17%
Subpulmonary RV failure	26 ms	6%	NYHA 2.4 to 1.6	N/A
Systemic RV failure	15–38 ms	7%–14%	25%–100%	14%–75%
Single-ventricle failure	13–45 ms	0%–10%	29%–91%	9%–71%

EF: ejection fraction; CRT: cardiac resynchronization therapy; DCM: dilated cardiomyopathy; LBBB: left bundle branch block; NYHA: New York Heart Association; RV: right ventricular; N/A: not applicable.
